# Characterization of Proteins Regulated by Androgen and Protein Kinase a Signaling in VCaP Prostate Cancer Cells

**DOI:** 10.3390/biomedicines9101404

**Published:** 2021-10-06

**Authors:** Hye-Jin You, Byong-Chul You, Jong-Kwang Kim, Jae-Min Park, Bo-Seul Song, Jae-Kyung Myung

**Affiliations:** 1 Division of Translational Science, National Cancer Center, 323 Ilsan-ro, Ilsandong-gu, Goyang-si 10408, Korea; hjyou@ncc.re.kr (H.-J.Y.); yoo_akh@ncc.re.kr (B.-C.Y.); 2 Department of Cancer Biomedical Science, National Cancer Center-Graduate School of Cancer Science and Policy, 323 Ilsan-ro, Ilsandong-gu, Goyang-si 10408, Korea; jam9192@naver.com (J.-M.P.); mawsonyya@naver.com (B.-S.S.); 3 Research Core Center, National Cancer Center, 323 Ilsan-ro, Ilsandong-gu, Goyang-si 10408, Korea; jkkim.gensdei@gmail.com

**Keywords:** castration resistant prostate cancer, proteome, metabolites, signaling pathway, androgen

## Abstract

Androgen signaling via the androgen receptor (AR) is involved in normal prostate development and prostate cancer progression. In addition to androgen binding, a variety of protein kinases, including cyclic AMP-dependent protein kinase A (PKA), can activate the AR. Although hormone deprivation, especially that of androgen, continues to be an important strategy for treating prostate cancer patients, the disease ultimately progresses to castration-resistant prostate cancer (CRPC), despite a continuous hormone-deprived environment. To date, it remains unclear which pathways in this progression are active and targetable. Here, we performed a proteomic analysis of VCaP cells stimulated with androgen or forskolin to identify proteins specific for androgen-induced and androgen-bypassing signaling, respectively. Patterns of differentially expressed proteins were quantified, and eight proteins showing significant changes in expression were identified. Functional information, including a Gene Ontology analysis, revealed that most of these proteins are involved in metabolic processes and are associated with cancer. The mRNA and protein expression of selected proteins was validated, and functional correlations of identified proteins with signaling in VCaP cells were assessed by measuring metabolites related to each enzyme. These analyses offered new clues regarding effector molecules involved in prostate cancer development, insights that are supported by the demonstration of increased expression levels of the eight identified proteins in prostate cancer patients and assessments of the progression-free interval. Taken together, our findings show that aberrant levels of eight proteins reflect molecular changes that are significantly regulated by androgen and/or PKA signaling pathways, suggesting possible molecular mechanisms of CRPC.

## 1. Introduction

Prostate cancer is the most common cancer and the second leading cause of cancer death among men. Between 1973 and 2013, prostate cancer incidence rates increased in all parts of the world [1]. When detected early, 70–80% of prostate cancer cases can be completely cured through surgery and castration therapy. Hormone (androgen) deprivation is also an important approach for treating prostate cancer patients. However, after 6 to 36 months of androgen-deprivation therapy (ADT), prostate cancer recurs in 20% of cases and develops into intractable castration-resistant prostate cancer (CRPC) [2,3], implying the involvement of other androgen-independent signaling pathways in CRPC progression.

Studies undertaken to understand the mechanism of CRPC development have indicated the active involvement of the androgen axis in CRPC growth [3,4,5,6]. Research reported that intratumoral androgens are synthesized in situ and that their metabolism contributes to CRPC [7,8,9,10,11,12,13]. Mutations, alternative splicing, and other alterations of the androgen receptor (AR) gene have been proposed to affect signaling within CRPC [14,15,16,17,18,19], suggesting the involvement of complex signaling pathways.

Testosterone, the main hormone involved in early prostate development, can be converted to dihydrotestosterone (DHT) via 5 alpha-reductase [20,21]. DHT is responsible for activating androgen signaling and facilitating continued AR signaling in the progression to CRPC [22]. The AR is a member of the steroid receptor family of transcription factors, which share structurally conserved domains, including a DNA-binding domain (DBD), a ligand-binding domain (LBD), an N-terminal domain (NTD), and a hinge region that contains a nuclear localization sequence. 

Androgen-dependent prostate cancer can be treated through targeting androgen synthesis or the AR ligand-binding domain [23,24]. However, CRPC is almost impossible to treat because of the operation of androgen-independent mechanism involving a variety of protein kinases, including cyclic AMP-dependent protein kinase A (PKA) and ligand binding domain-deleted AR variants (AR-Vs) [25]. 

PKA is activated by the second messenger, cAMP [26,27,28], that are necessary for the proper biological response of cells to hormones and other extracellular signals [29]. This PKA-signaling pathway can be stimulated by the synthetic compound forskolin (FSK), which acts directly on adenylate cyclase to increase intracellular levels of cAMP, thereby, inducing PKA-dependent AR activation [27,30,31,32].

The molecular expression profiling of prostate cancer cells has led to the identification of expression patterns that are associated with specific phenotypes and prognosis. Differential expression has been determined in prostate cancer cells stimulated with androgen-induced or PKA-induced AR signaling by treating cells with DHT or FSK, respectively [33,34,35]. 

To date, there are no protein expression profiling of androgen- and PKA-induced VCaP cells, which are one of the most representative CRPC models with amphicrine feature [36]. Here, using two-dimensional electrophoresis (2DE), we identified differences in proteomes between androgen (DHT)- and PKA (FSK)-stimulated VCaP prostate cancer cells and control (untreated) VCaP cells. Ultimately, the identified significant differences in proteins induced by DHT and FSK treatment may provide insights into prostate cancer progression and help guide the development of new CRPC treatments.

## 2. Materials and Methods

### 2.1. Cell Culture and Treatment

VCaP cells were obtained from American Type Culture Collection (ATCC, Rockville, MD, USA). Cells were previously authenticated by the NCC Omics Core facility (Perkin Elmer, Waltham, MA, USA) using the short-tandem repeat (STR) polymerase chain reaction (PCR) method. Cells were cultured in Dulbecco’s Modified Eagle’s medium (DMEM; Sigma-Aldrich, St. Louis, MO, USA) containing 10% fetal bovine serum (FBS; Gibco, Carlsbad, CA, USA), 100 μg/mL streptomycin, and 100 U/mL penicillin (Gibco). Cells were incubated at 37 °C in a humidified 5% CO_2_ environment. VCaP cells were serum-starved and treated with 10 nM DHT or 1 μM FSK for 3 h.

### 2.2. Protein Sample Preparation and 2DE

Proteins were extracted from cells using a urea lysis buffer (7 M urea, 2 M thiourea, 65 mM CHAPS, 0.5 M EDTA, 50 mM Tris, 0.01% BPB, and 65 mM DTT) supplemented with protease inhibitors (Roche), 200 mM PMSF (phenylmethylsulfonyl fluoride), and ampholytes. Cell lysates were desalted and concentrated using Amicon ultra centrifugal filters (Merck Millipore, Darmstadt, Germany), and the resulting protein concentration was measured using a Bradford protein assay kit (Bio-Rad, Hercules, CA, USA) according to the manufacturer’s instructions.

Proteins were resolved by 2DE, which separates proteins based on isoelectric point (first dimension) and size (second dimension). For isoelectric focusing (IEF), each protein sample was loaded on an IPG strip (pH 3–10 NL; 130 mm × 3 mm × 0.5 mm, GE Healthcare), after which the strip was rehydrated for 18 h. After performing the IEF electrophoresis step for a total of 45,000 Vhrs, the IPG strip was first soaked in equilibration buffer consisting of 0.5 M Tris pH 8.8, 6 M urea, 2% SDS, and 30% glycerol containing 100 mM DTT for 15 min, and then in equilibration buffer containing 110 mM iodoacetamide (IAA) for 15 min. For the second dimension, proteins were separated using sodium dodecyl sulfate-polyacrylamide gel electrophoresis (SDS-PAGE). Colloidal Coomassie blue staining was used to visualize the separated protein spots.

### 2.3. Protein Quantification and Identification

A total of nine stained gels were quantified using the Delta2D software according to the manufacturer’s instructions. *p*-values < 0.05 (Student’s *t*-test) were taken as indicating a significant difference in expression. Among the matched protein spots (*n* = 113), those with significant quantitative difference were selected from each comparative analysis and identified (Control vs. DHT or FSK).

Proteins were identified by excising protein spots from 2DE gels for in-gel tryptic digestion using an in-gel tryptic digestion kit (Thermo Fisher Scientific, Rockford, IL, USA), according to the manufacturer’s instructions. Briefly, excised gels were destained, reduced with TCEP (tris [2-carboxyethyl] phosphine), and alkylated with iodoacetamide (IAA). The alkylated gel pieces were dehydrated in 100% acetonitrile (ACN) and digested with mass spectrometry (MS) grade trypsin for 12 h at 30 °C. Digested peptides were dried by evaporation using a vacuum concentrator and cleaned up for MS analysis using C18 spin columns (Thermo Fisher Scientific, Rockford, IL, USA).

Tryptic-digested peptides were analyzed using an Q Exactive hybrid quadrupole-orbitrap mass spectrometer (Thermo Fisher Scientific, Rockford, IL, USA) coupled to an Ultimate 3000 RSLC nano system (Thermo Fisher Scientific, Rockford, IL, USA). The tryptic peptides were loaded onto a trap column (100 μm × 2 cm) packed with Acclaim PepMap100 C18 resin, and eluted with a linear 5% to 30% gradient of solvent B (0.1% formic acid in ACN) for 120 min at a flow rate of 300 nL/min. 

The eluted peptides, separated using an EASY-Spray analytical column (75 μm × 15 cm; Thermo Fisher Scientific), were sprayed into a nano-ESI source at an electrospray voltage of 2.4 kV. Full MS scans were acquired over the m/z 300–2000 range with a mass resolution of 70,000 (at *m*/*z* 200) using a Q Exactive Orbitrap mass analyzer operated using the top 10 data-dependent method. The AGC target value was 1.00 × 10^6^. The ten most-intense peaks with a charge state ≥2 were fragmented in the higher-energy collisional dissociation (HCD) cell with a normalized collision energy of 25%, and tandem mass spectra were acquired in the Orbitrap mass analyzer with a mass resolution of 17,500 at *m*/*z* 200.

Database searching of all raw data files was performed using Proteome Discoverer software (Thermo Fisher Scientific, Rockford, IL, USA). The UniProt database was searched using SEQUEST-HT. The false-discovery rate (FDR) for peptide identification was evaluated by searching raw data against the corresponding reversed database. Database searching parameters included the following: up to two missed cleavages allowed for full tryptic digestion; precursor ion mass tolerance, 10 ppm; fragment ion mass tolerance, 0.02 Da; fixed modification for carbamidomethyl cysteine; and variable modifications for methionine oxidation and N/Q deamination. An FDR less than 1% was obtained at the peptide level, and peptides were filtered with high confidence.

### 2.4. Metabolite Sample Preparation and Identification

Frozen pellets of cells treated with R1811 (10 nM) or FSK (1 μM) for 3 and 24 h were thawed and kept on ice. The thawed pellets were suspended in 500 μL of methanol, mixed by vortexing, and subsequently subjected to three freeze/thaw cycles. After centrifuging at 800× *g* for 1 min, the supernatants were collected and transferred to new tubes. Next, the pellets remaining after the previous centrifugation step were suspended in 250 μL of water, mixed by vortexing, and subjected to the same freeze/thaw process described above. All resulting supernatants were collected and dried using a concentrator.

The dried samples were reconstituted in 0.1% formic acid and applied to a Liquid Chromatograph-Tandem Mass Spectrometer (LC-MS/MS) consisting of an ExionLC system (AB Sciex, Foster City, CA, USA) and triple quad 5500+ system. Sample separation was achieved using Ultra high-performance LC with an Atlantis T3 column (3 μm, 2.1 mm × 10 mm; Waters, Milford, MA USA). A targeted profiling approach was applied using multiple reaction monitoring (MRM) of the MS system with reference standards for NADH, 4-hydroxynonenal, ATP, and lactic acid (Sigma-Aldrich). The following parameters were used for the MS system: turbo ion-spray voltage, +5500 v; temperature, 500 °C; curtain gas, 40 psi; CAD gas, 12 psi; and gas 1 and 2, 50 psi.

### 2.5. Western Blot Analysis

Treated VCaP cells were washed once with cold phosphate-buffered saline (PBS) and lysed by incubating in radioimmunoprecipitation (RIPA) lysis buffer (20 mM Tris-HCl pH 7.5, 150 mM NaCl, 1% NP-40, 0.5% sodium deoxycholate, 1 mM EDTA, 10 mg/mL leupeptin, 10 mg/mL aprotinin, 2 mM NaVO_4_, 10 mM β-glycerophosphate, and 1 tablet protease inhibitor) for 1 h on ice. The lysates were centrifuged at 14,000 rpm for 30 min at 4 °C, and the supernatants were collected. The protein concentrations were determined using an enhanced BCA protein assay kit (Thermo Fisher Scientific, Rockford, IL, USA). Equal amounts of total protein from each sample were resolved by SDS-PAGE on 10% gels and transferred to PVDF (polyvinylidene difluoride) membranes. 

After blocking with 50% Odyssey Blocking Buffer in PBS containing 0.05% Tween-20 (PBS-T) for 1 h at room temperature, the membrane was incubated with anti-TUFM (Thermo Fisher Scientific, Rockford, IL, USA), anti-OXCT1 (Novus Biologicals), anti-ACPP (Novus Biologicals), or anti-LDHB (Abcam) primary antibody overnight at 4 °C. The membrane was then washed four times with PBS-T for 5 min each and incubated with the appropriate secondary antibody (Santa Cruz Biotechnology) for 1 h at room temperature. Specific protein bands were detected using the ECL^TM^ Prime western blotting detection reagent (GE Healthcare Life Sciences).

### 2.6. Quantitative PCR Analysis

The total RNA was extracted from androgen- or FSK-treated VCaP cells using the TRIzol Reagent (Favorgen Biotech Corp, Ping-Tung, Taiwan). A total of 1 μg of RNA was reverse transcribed into cDNA using an AmpiGene cDNA Synthesis Kit (Enzo Biochem, New York, NY, USA). The resulting cDNA was quantified by real-time quantitative PCR using amfiSure qGreen Q-PCR master mix (GenDEPOT, Barker, TX, USA) and primers synthesized by Macrogen (Seoul, South Korea) as indicated in Table S1. The expression of target mRNAs was quantified using the 2^−ΔΔCT^ method and normalized to the levels of GAPDH, which was used as an internal control.

### 2.7. Analysis of the Progression-Free Interval and Gene Expression

The RNA-seq TPM gene expression data for prostate cancer cell lines were downloaded from Dependency Map portal (https://depmap.org, 7 September 2021), and gene expression profiles and clinical data were downloaded from The Cancer Genome Atlas (TCGA). Clinical outcomes for prostate adenocarcinoma (PRAD) of the TCGA study with Gleason score ≥6 and the pooled normal and GTEx normal were used for comparison of gene expression pattern in tumor and normal and survival analyses [37]. 

In this latter study, the progression-free interval was recommended instead of the overall or disease-free survival since there were only 10 overall survival events out of 500 cases of PRAD in the TCGA study [37]. For comparisons of survival among patient groups, we divided samples according to the median mRNA expression level and included only samples that were greater than the upper quartile and lower than the lower quartile. Kaplan–Meier curves of two groups were compared using a log-rank test (*p*-value < 0.05) in the R survival package, survdiff, with the default parameters.

## 3. Results

### 3.1. Androgen- and PKA Signaling-Induced Changes in the Proteomic Profile of VCaP Cells and Identification of Differentially Expressed Proteins

AR signaling is active in CRPC regardless of androgen status. Importantly, however, the distinctive factors that mediate androgen-dependent and -independent AR signaling remain to be elucidated. To identify distinct proteins that mediate androgen-induced and androgen-independent signaling pathways, we first activated signaling pathways in VCaP cells by stimulation with DHT or FSK, respectively and then assessed protein expression using 2DE analysis. Differentially expressed proteins between proteomes obtained from control cells and DHT- or FSK-treated cells were assessed by performing a spot-by-spot analysis of the intensity of each protein spot in 2DE gels. For precise and reproducible experimental data, we performed three biological replicates in triplicate (Figure S1).

**Figure 1:**
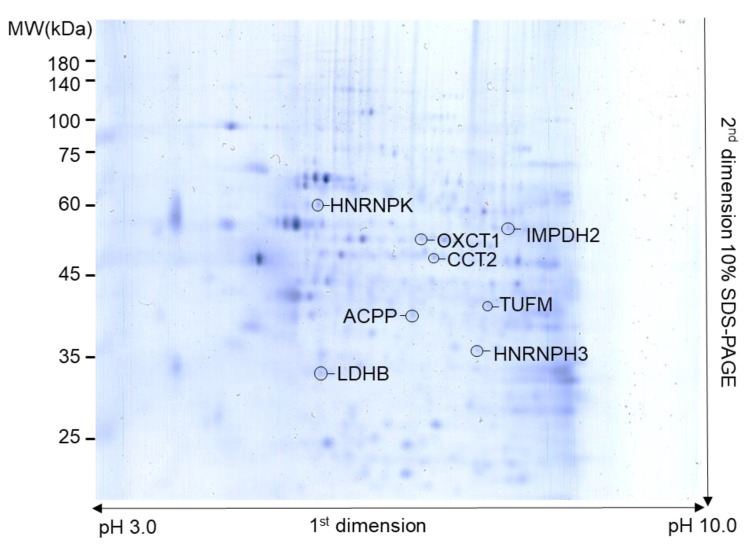
2DE analysis and identification of protein spots showing significant changes in expression between the control group and DHT-or FSK-treated groups. Representative gel showing eight protein spots with significant changes in expression (density) among DHT-, FSK-treated groups, and the control group as well as the identification of proteins by MS analysis.

**Figure 2:**
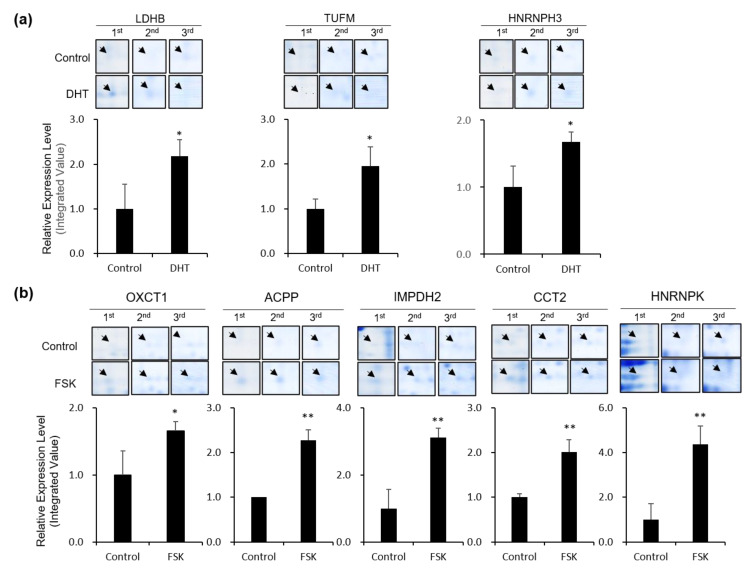
Comparative expression levels of the identified protein spots. Protein spots and the relative expression levels of proteins regulated by DHT (**a**) and FSK (**b**) from 2DE analysis. Significantly regulated proteins exhibiting between-group changes of 1.5-fold or more (* *p* < 0.05, ** *p* < 0.01) are presented. The values were calculated based on spot densities obtained using PDQuest. The data obtained from the mean ± standard deviation (SD) of three independent experiments are presented as fold changes.

Overall, 113 proteins spots with different expression levels were detected between the control and DHT or FSK group, of which eight ultimately exhibited significant differences in density (>1.5-fold changes in expression with *p* < 0.05). All eight differentially expressed proteins, shown in a representative 2DE gel (Figure 1), exhibited a clear and significant increase in response to DHT or FSK in three repeat experiments (Figure 2 and Table 1). Subsequent MS analyses unambiguously assigned identities to individual proteins. This analysis successfully identified three proteins that were altered in VCaP cells in response to DHT (androgen-induced signaling): LDHB (L-lactate dehydrogenase B chain), TUFM (elongation factor Tu), and HNRNPH3 (heterogeneous nuclear ribonucleoprotein H3). 

Five proteins—OXCT1 (succinyl-CoA:3-ketoacid coenzyme A transferase 1), ACPP (prostatic acid phosphatase), IMPDH2 (inosine-5′-monophosphate dehydrogenase 2), CCT2 (T-complex protein 1subunit beta), and HNRNPK (heterogeneous nuclear ribonucleoprotein K), were found to be specifically increased in VCaP cells in response to FSK. Representative MS/MS spectra of protein spots are shown in Figure S2, and the number of unique peptides, molecular weights, and isoelectric points for each protein, derived from MS analysis, are shown in Table S2.

To investigate the functional role of identified proteins, we performed a Gene Ontology (Go) analysis of their cellular localization (cellular component) and biological role (biological process). This information is summarized in Table S2. Interestingly, this analysis revealed that all identified proteins are involved in metabolic processes. Notably, metabolic reprogramming is known to be associated with re/activation and antagonism of AR signaling, which, in turn, drives CRPC progression [38]. Further metabolic process information was obtained for major molecules associated with the identified proteins (Please see Section 3.3).

### 3.2. Validation of Androgen- and PKA Signaling–Specific Differentially Expressed Proteins

**Figure 3:**
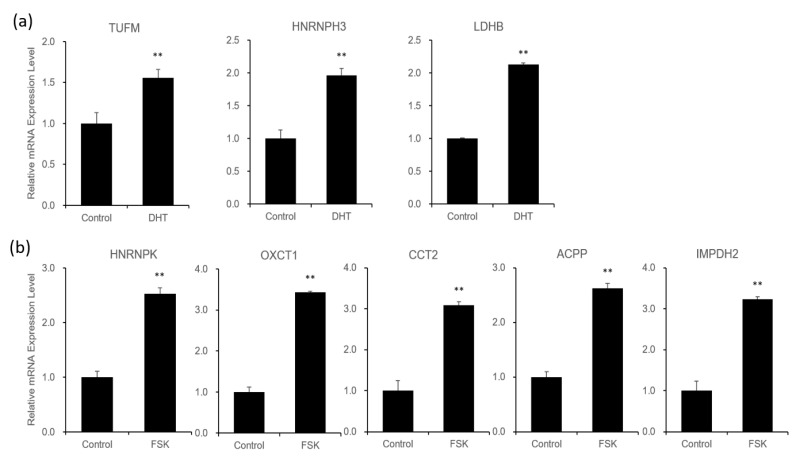
Relative mRNA expression levels of identified proteins that are significantly induced by androgen and PKA signaling pathways. Changes in the mRNA level of proteins significantly modulated by DHT (**a**) or FSK (**b**), as determined by qRT-PCR analysis. All data obtained from the means ± SD of three independent experiments are presented as fold changes (** *p* < 0.01).

Next, using quantitative RT-PCR, we further confirmed the DHT- or FSK-induced increases in expression of all eight proteins at the mRNA level, suggesting a pathway-specific role of DHT- and FSK-induced proteins in VCaP cells (Figure 3).

**Figure 4:**
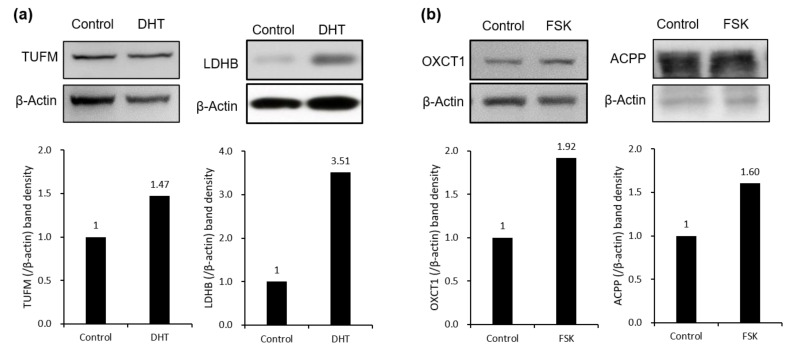
Confirmation of significant changes in the protein expression level. The levels of proteins found to be significantly regulated by DHT (**a**) and FSK (**b**) in our 2DE analysis were confirmed by western blot analysis. Results are the representative of three independent experiments and fold change of expression was labeled.

To extend our knowledge of the specific expression of eight proteins in a prostate cancer cell with amphicrine features, like VCaP, we determined the endogenous expression level of proteins in five different prostate cancer cell lines from the Dependency Map portal (https://depmap.org, accessed on 7 September 2021) along with the expression of AR and neuroendocrine biomarker, synaptophysin (SYP), which shows an amphicrine feature [36]. All eight proteins were observed in VCaP cells as well as other prostate cancer LNCaP, 22RV1, MDAPCA2B, and PC3 cells with a different expression level (Figure S3). TUFM and ACPP, which are frequently associated with prostate cancer, and two metabolic enzymes, LDHB and OXCT1, were further confirmed at the protein level in DHT (androgen)- and FSK (PKA signaling)-stimulated VCaP cells by immunoblotting (Figure 4a,b).

TUFM is a key factor in the translational expression of mitochondrial DNA, playing an important role in the control of mitochondrial function, whereas ACPP is a glycoprotein that is mainly synthesized and secreted by glandular epithelial cells of the prostate. The expression of both ACPP and PSA mRNA is significantly elevated in prostatic carcinoma compared with that in benign prostatic hyperplasia [39,40,41]. In addition, TUFM is upregulated at the protein level in prostate cancer [42,43], and ACPP has been used as a diagnostic and prognostic marker together with prostate-specific antigen (PSA) for prostate cancer.

LDHB, induced by androgen-specific signaling, is a well-known metabolic enzyme involved in lactate production, which leads to bypassing of oxidative phosphorylation, especially in glycolic cancer cells [44,45]. It has been proposed that pancreatic cancer [46] and breast cancer [47] patients with lower LDHB expression are more likely to show positive responses to treatment, and LDHB has frequently been proposed as a diagnostic and prognostic marker in prostate cancer [48,49]. In this study, we found increased expression of LDHB in androgen-stimulated VCaP cells (Figure 4a, right), supporting the prognostic and diagnostic value of LDHB as well as its role as a therapeutic target in prostate cancer.

OXCT1, an enzyme that catalyzes the reversible transfer of CoA from succinyl-CoA to acetoacetate in mitochondrial membranes [50], is considered a therapeutic target in cancer by virtue of its regulation of ketone bodies [51]. OXCT1 expression is increased in LNCaP-SF cells, an androgen-independent LNCaP cell line derivative, as well as in high-grade prostate cancers relative to normal and low-grade samples [52]. In this study, OXCT1 expression was induced by PKA signaling at both the mRNA and protein levels in VCaP cells (Figure 3b and Figure 4b). As is the case in androgen-independent cell lines, OXCT1 is thought to contribute to the metabolic processing involved in the development of advanced prostate cancer stages.

### 3.3. Androgen- and PKA Signaling-Induced Metabolic Alterations in VCaP Cells

**Figure 5:**
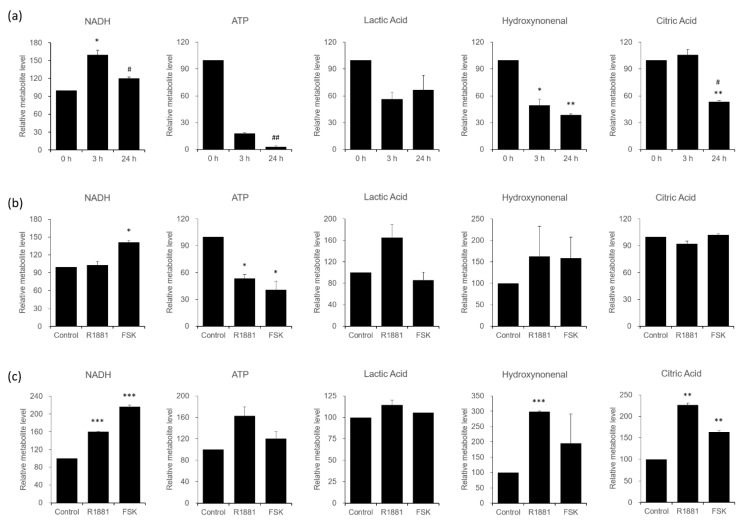
Determination of the differential expression levels of metabolites, NADH, ATP, lactic acid, hydroxynonenal, and citric acid in VCaP cells. Metabolite concentrations modulated by R1881 and FSK were measured in VCaP cells at 3 and 24 h. (**a**) The time course of changes in metabolites, measured in serum-starved VCaP cells. (**b**) Changes in metabolites associated with androgen or PKA signaling pathways, measured at 3 h. (**c**) Changes in metabolites associated with androgen or PKA signaling pathways, measured at 24 h. Statistical significance is indicated as follows: (**a**): * *p* < 0.05, ** *p* < 0.01 when compared with non-starved control group, ^#^ *p* < 0.05, ^##^ *p* < 0.01 when compared with 3-h serum-starved group. (**b**): * *p* < 0.05 when compared with untreated control group. (**c**): ** *p* < 0.01, *** *p* < 0.001 when compared with the untreated control group.

Some of the differentially expressed proteins identified in VCaP cells are involved in the metabolism, including LDHB, which was increased in androgen-induced signaling only, and IMPDH2 and OXCT1, which were increased in FSK-induced signaling only, leading us to further validate signaling-specific metabolic alterations. To this end, we treated VCaP cells with androgen (10 nM R1881) or FSK (1 μM) for 3 or 24 h, and after harvesting cells, we measured the metabolites by MS analysis (Figure 5).

Dysregulated metabolism for increased energy production to provide enough proliferation and growth is one of the hallmarks of cancer cells. Prostate cancer has a unique metabolic feature with specific metabolic and energetic phenotypes according to the stage of cancer progression [53], such as the absence of the Warburg effect observed in primary prostate cancer. The understanding of the relationship between these distinctive metabolic features and AR signaling in PCa is crucial [38]. 

Serum-starved VCaP cells showed a gradual decrease over time in the intracellular concentrations of ATP ([ATP]_i_), lactic acid ([lactic acid]_i_), hydroxynonenal ([hydroxynonenal]_i_), and citric acid ([citric acid]_i_), and an increase in NADH concentration in the cell ([NADH]_i_) after treatment for 3 and 24 h compared with the pretreatment values (t_0_) (Figure 5a). Both androgen- and FSK-induced signaling reduced [ATP]_i_ and increased [hydroxynonenal]_i_ at 3 h (Figure 5b); in contrast, [lactic acid]_i_ was increased at 3 h and came back to a similar level of control at 24 h only in androgen-stimulated cells, while [NADH]_i_ was increased only in FSK-stimulated cells at 3 h. 

Interestingly, [hydroxynonenal]_i_, [ATP]_i_, and [citric acid]_i_ were increased in androgen-stimulated cells at 24 h (Figure 5c), which implies a role of androgen-induced signaling on metabolic pathways through proteins, including LDHB. Alteration of [lactic acid]_i_, [ATP]_i_, [NADH]_i_, and [citric acid]_i_ in a time course manner, suggested a role of androgen on energy metabolism, particularly ATP synthesis, through oxidative phosphorylation in androgen-induced signaling. [ATP]_i_, [NADH]_i_, and [hydroxynonenal]_i_ showed a distinct energy metabolic regulation through fatty acid synthesis pathways in FSK-induced VCaP cells.

In terms of the energy metabolism, androgen signaling might be beneficial for the efficiency of ATP generation through two different pathways: oxidative phosphorylation, mediated by androgen binding, and the fatty acid synthetic pathways, specifically facilitated by FSK-induced, PKA-mediated activation. The metabolism in CRPC is mainly studied from the standpoint of overcoming androgen castration. Here, we provide evidence for distinct pathways involved in the acquisition of androgen signaling in VCaP cells: direct AR activation by androgen binding and indirect AR activation via PKA.

### 3.4. Clinical Correlations of Proteins That Are Significantly Altered by Androgen- or PKA Signaling Pathways

Androgen directly binds to the AR, a nuclear receptor that signals by regulating androgen-response element-dependent gene expression. In our study, eight proteins were altered within 3 h of DHT or FSK stimulation, exhibiting specific responses to the two agents. An assessment of alterations in metabolites in response to R1881 and FSK exposure showed significant common effects on [ATP]_i_ but distinct effects on a few metabolites. To gain insight into whether these eight proteins are involved in prostate cancer progression and malignancy, we obtained prostate cancer expression data from the TCGA database (http://www.cbioportal.org, accessed on 12 Feburary 2021) and further analyzed the correlation between progression-free interval and the expression of each protein. 

**Figure 6:**
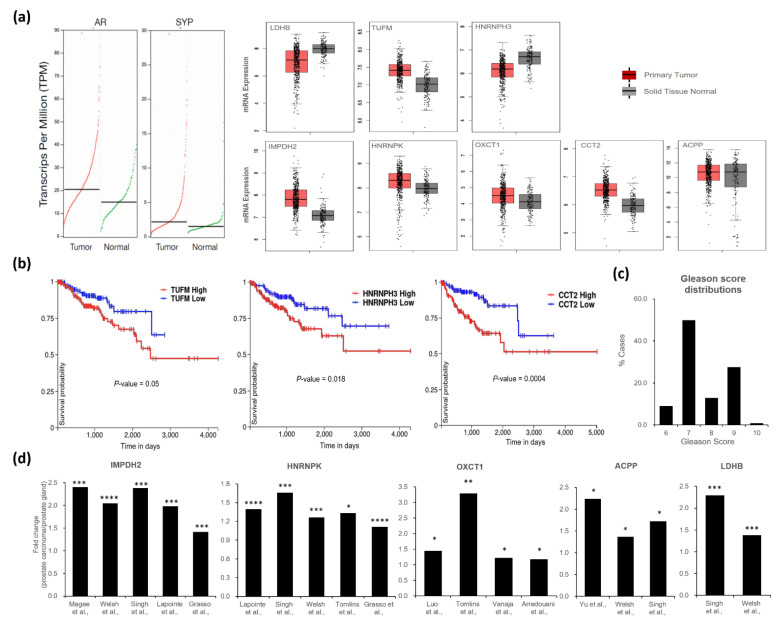
Protein expression and progression-free interval in prostate cancer patients. (**a**) Dot plots show the profiling of AR and SYP gene expression across tumor and paired normal samples, with each dot representing a distinct tumor or normal samples (left), and the relative expression of eight genes (right) was represented in normal tissues versus tumor tissues with a Gleason score ≥ 6. (**b**) Kaplan-Meier curves show that changes in the mRNA expression of DHT- and FSK-regulated proteins are associated with clinical outcomes in samples from the TCGA PRAD database (*n* = 550; log-rank *p*-value < 0.05). (**c**) Gleason score distribution was represented from patients used in this study. (**d**) Differences in gene expression were quantified as fold changes in prostate carcinomas, including hormone refractory prostate cancer and metastatic prostate cancer samples compared with prostate gland samples from various datasets [57,58,59,60,61,62,63,64,65,66] (* *p* < 0.05, ** *p* < 0.01, *** *p* < 0.001, **** *p* < 0.0001).

This analysis was conducted on a cohort in which all tumors had a Gleason Score of 6 or higher demonstrating the worse and poor prognosis [54,55,56]. VCaP cells display an amphicrine profile, which is the co-expression of the AR, AR target genes and neuroendocrine (NE) genes and AR and classical NE biomarker, SYP [36]. Thus, AR and SYP were included for the expression analysis along with eight proteins. As shown in Figure 6a, changes of expression levels of eight proteins were observed in tumors compared with normal tissue, and the expression levels of AR and SYP were increased implying that clinical samples used in TCGA analysis have an amphicrine phenotype. 

In addition, the expression levels of three proteins—TUFM, and HNRNPH3 from the DHT-specific proteome, and CCT2 from the FSK-specific proteome—were related to the progression-free interval in prostate cancer patients (Figure 6b). The increased expression levels of TUFM, HNRNPH3, and CCT2 were significantly correlated with survival without progression, suggesting a possible role for each protein in CRPC development especially with a higher Gleason score (Figure 6b,c). 

In addition to TCGA data analysis, we also analyzed the expression levels of the five proteins from the DHT-specific protein, LDHB as well as FSK-specific proteins, IMPDH2, HNRNPK, OXCT1, and ACPP in protein carcinomas, including hormone refractory prostate cancer and metastatic prostate cancer samples in several publicly available datasets. Interestingly, these proteins showed significantly greater expression in prostate tumor tissues than in normal or adjacent normal tissues (Figure 6d), suggesting that signaling-specific proteins identified in VCaP cells are relevant in the context of advanced prostate cancer.

## 4. Discussion

In CRPC, one mechanism of resistance against hormone deprivation and progression is thought to be the expression of truncated AR variants. These AR variants lack a C-terminal domain, thus, resulting in androgen-independent signaling [19,67]. Using LNCaP cells, which express mutant AR, we stimulated androgen-induced or PKA-induced AR signaling by treating cells with DHT or FSK, respectively, and assessed differences in the proteomes between the two treatments using 2DE [35]. Here, we have studied differential proteome expression using VCaP cells, which express both wild-type AR and AR splicing variants. 

This analysis identified eight signaling-specific proteins, three from the androgen-specific proteome and five from the PKA-induced proteome, all of which were subsequently validated in MS analyses and cell-based studies (Figure 2, Figure 3 and Figure 4). Interestingly, most proteins that showed significantly different changes in expression are known to be involved in metabolic processes. A further investigation of the involvement those of proteins in the metabolic transformation, which plays an important role in prostate cancer progression, revealed alterations in levels of the metabolites, ATP, NADH, lactic acid, hydroxynonenal, and citric acid in response to R1881 or FSK. Some metabolites were altered in common, whereas others were altered in an agonist-specific manner (Figure 5).

Lactate dehydrogenase (LDH) is the primary metabolic enzyme that converts pyruvate to lactate, and vice versa, making it an important player in the cancer metabolism. LDHB is found at the highest densities in mitochondria; and, in normoxic cells, mitochondrial LDHB converts lactate to pyruvate. This lactate-derived pyruvate can then be used as fuel for the TCA cycle, oxidative phosphorylation, and mitochondrial respiration [68,69].

Although the absence of the LDHB was not found in LNCaP but in LNCaP-LN3 cells at the protein and mRNA level [70], and the loss of LDHB increased the tumorigenicity of prostate cancer cells [71], it has been shown that increased LDHB activity and the Warburg effect are required for tumor progression and metastases in a preclinical model of prostate cancer [72]. Consistent with this, LDHB expression is highly elevated in lung cancer [73] and breast cancer [47,74]. Under acidic conditions with high lactate, androgen may induce an increase in LDHB in VCaP cells, resulting in a decrease in lactic acid and an increase in pyruvate for oxidative phosphorylation and ATP generation; NAD is also increased under these conditions, leading to an increase in NADH (Figure 5). In fact, LDHB was shown to control tumor progression and cancer cell proliferation through modulation of lysosome activity and autophagy [75].

We also observed upregulated IMPDH2 protein (Figure 2) and increased NADH (Figure 5) in FSK-stimulated VCaP cells. IMP dehydrogenase (IMPDH) catalyzes the oxidation of IMP to XMP, with the concomitant reduction of NAD to NADH, playing a role as a nucleotide biosynthetic enzyme; it also acts as a transcription factor to regulate proliferation-associated genes [76,77]. Interestingly, [NADH]_i_ was higher in FSK-stimulated cells than in androgen-stimulated cells at both 3 and 24 h (Figure 5), whereas [hydroxynonenal]_i_ was less at 24 h in FSK-stimulated cells than in androgen-stimulated cells, implying a role for NADH in the peroxidation of lipids for cellular energy metabolism and redox balance.

Importantly, candidate proteins, IMPDH2, HNRNPK, OXCT1, ACPP, and LDHB, were highly expressed in progressive prostate cancer patients (Figure 6d, and the elevated expression of TUFM, HNRNPH3, and CCT2 was significantly associated with progression-free interval in prostate cancer patients diagnosed with a Gleason Score 6 or higher (Figure 6b, supporting the inference that the identified proteins might contribute to prostate cancer progression. In addition to previous molecular studies on the enhanced expression of IMPDH2 [78,79,80], HNRNPK [81], OXCT1 [52], ACPP [39,40,41], LDHB [82], TUFM [42,43], HNRNPH3 [83], and CCT2 ([84,85,86], dysregulated expression of those proteins may be useful for clinicopathological features of prostate cancer patients. In terms of treatment resistance, metastatic CRPC has been studied for better therapeutic options and overcoming the resistance. 

In one of these approaches, Dr. Morrissey and Dr. Nelson and colleagues characterized metastatic CRPC and cell lines into five phenotypes depending on the AR or NE genes [87,88]. According to their phenotypes, VCaP cell lines are considered as amphicrine (AMPC) expressing both AR and NE genes, which are used to define the molecular characteristics of samples used for expression analysis in cell lines and clinical samples (Figure 6a,b and Figure S3). Here, we report eight proteins altered by androgen-induced or PKA-induced signaling; however, the detailed mechanism is not clear, and further investigation will be required to elucidate how they contribute to AMPC phenotype and drug response in prostate cancer cells.

Taken together, our findings highlight eight proteins specific to androgen or PKA signaling proteomes that were significantly regulated and validated in cells and tissues. In addition, we further identified a significant association of candidate proteins with metabolic processes. Aberrant protein levels may reflect molecular changes regulated by androgen and/or PKA signaling pathways in the context of AR signaling. Thus, our findings provide helpful insights into prostate cancer progression generally and the relationship between intracellular factors and AR signaling cascades, specifically.

## Figures and Tables

**Table 1:** The average density values of proteins that showed significant changes in expression in response to DHT or FSK.

Comparison	Protein Name	Mean		SD *		Ratio	*p*-Value
		Control	Treatment	Control	Treatment		
Control vs. DHT	LDHB	15.838	34.567	8.736	5.858	2.183	0.037
	TUFM	7.120	13.901	1.554	3.078	1.952	0.027
	HNRNPH3	10.584	17.706	3.311	1.570	1.679	0.027
Control vs. FSK	OXCT1	6.773	11.221	2.447	0.929	1.657	0.042
	ACPP	12.637	28.735	2.900	3.534	2.274	0.004
	IMPDH2	5.937	18.424	3.404	1.666	3.103	0.005
	CCT2	9.235	18.633	0.709	2.483	2.018	0.003
	HNRNPK	7.573	33.052	5.482	6.157	4.365	0.006

* SD, Standard Deviation.

## Supplementary Materials

The following are available online at https://www.mdpi.com/article/10.3390/biomedicines9101404/s1, Figure S1: 2DE analysis of proteins from VCaP cells. Proteome analysis of VCaP prostate cancer cells treated with androgen (10 nM DHT) or forskolin (1 µM FSK) by 2DE analysis are represented. Proteins were resolved by IEF over the pI range 3–10, followed by 10% SDS-PAGE, and visualized with coomassie colloidal blue staining in triplicate. Figure S2: Representative MS/MS spectra of proteins identified using SEQUEST-HT. The representative spectrum was represented from the identified peptide ELLTEFGYK corresponding to TUFM, VHIDIGADGR corresponding to HNRNPH3, FIIPQIVK corresponding to the LDHB, DLAGSIIGK corresponding to HNRNPK, AGNVIFRK corresponding to OXCT1, LAVEAVLR corresponding to CCT2, FLNESYK corresponding to ACPP, and DRVRDVFEAK corresponding to IMPDH2. Figure S3. mRNA expression in different prostate cancer cell lines. The expression level of genes significantly regulated by androgen (LDHB, TUFM, and HNRNPH3) or forskolin (IMPDH2, HNRNPK, OXCT1, CCT2, and ACPP) was determined in LNCaP, VCaP, 22RV1, MDAPCA2B, and PC3 cells along with the expression of AR and the neuroendocrine biomarker, SYP. The expressions are Log2 transformed, using a pseudo-count of 1. Table S1: The oligonucleotide primers used in the study. Sequences of the oligonucleotide primers used in quantitative PCR analysis are shown. Table S2: List of proteins identified by MS analysis. Proteins with significant expression changes were identified by MS analysis and functional information including cellular components and the biological process is described.
