# Supplementary material for: Characterization of Proteins Regulated by Androgen and Protein Kinase a Signaling in VCaP Prostate Cancer Cells
Source: Biomedicines. 2021 Oct 6;9(10):1404. doi: 10.3390/biomedicines9101404 (PMC8533394; doi:10.3390/biomedicines9101404)
Supplement: Supplementary file 1 [file biomedicines-09-01404-s001.zip › Table S1 S2.pdf]

Table S1. Oligonucleotide primers used in the study.

Primer	Sequences	
TUFM	(forward)	5′ - GTT CTC CCT GAC TTG GGA CA -3′
	(reverse)	5′ - CCA GCG TGT TGG TGA CTA GA -3′
HNRNPH3	(forward)	5′ - GCT ATG GTG GAG CTG GTG AT -3′
	(reverse)	5′ - TCT GCT TCT CCT GTG GCT CT -3′
LDHB	(forward)	5′ - TGC TCT TGT GGA TGT TTT GG -3′
	(reverse)	5′ - GAC TCT CCC CTT CTT GCT GA -3′
HNRNPK	(forward)	5′ - TTC AGT CCC AGA CAG CAG TG -3′
	(reverse)	5′ - TCC ACA GCA GCA GAT TCG AG -3′
OXCT1	(forward)	5′ - GGG TCC ATA TCC ACG ACA AC -3′
	(reverse)	5′- CCA GTT AGC CAG GTC ACC AT -3′
CCT2	(forward)	5′ - GAA CAT GCG GAA AAG GAA AA - 3′
	(reverse)	5′ - AGA GGC AAT TTC ACC ACC TG - 3′
ACPP	(forward)	5′ - GAC GTT GAC CGG ACT TTG AT - 3′
	(reverse)	5′ - TTC TTG AAA ACG AGG GCA GT - 3′
IMPDH2	(forward)	5′ - CCG GAC AGA CCT GAA GAA GA -3′
	(reverse)	5′ - TGG GAA GAG TCC AAA ACC AC -3′

Table S2. List of proteins identified by MS

Treatment	Gene name	Acc. No. [1]	Protein name	MW [2] (KDa)	pI [3]	Cellular component	Biological process
DHT	TUFM	P49411	elongation factor Tu, mitochondrial	49.510	7.61	membrane; mitochondrion	metabolic process; response to stimulus
	HNRNPH3	P31942	Heterogeneous nuclear ribonucleoprotein H3	36.903	6.87	nucleus; spliceosomal complex	cell differentiation; metabolic process
	LDHB	P07195	L-lactate dehydrogenase B chain	36.615	6.05	cytoplasm; cytosol; membrane;mitochondrion	metabolic process
FSK	IMPDH2	P12268	inosine-5'-monophosphate dehydrogenase 2	55.770	6.90	cytoplasm; cytosol; extracellular; membrane; nucleus; organelle lumen	cell organization and biogenesis; cell proliferation; metabolic process; response to stimulus; transport
	HNRNPK	P61978	Heterogeneous nuclear ribonucleoprotein K	50.996	5.33	cytoplasm; membrane; nucleus; spliceosomal complex	metabolic process; regulation of biological process; response to stimulus
	OXCT1	P55809	Succinyl-CoA:3-ketoacid coenzyme A transferase 1, mitochondrial	56.122	7.46	mitochondrion; organelle lumen	metabolic process; regulation of biological process; response to stimulus
	CCT2	P78371	T-complex protein 1 subunit beta	57.452	6.46	cytoplasm; cytosol; extracellular; organelle lumen	cell organization and biogenesis; metabolic process; regulation of biological process; transport
	ACPP	P15309	Prostatic acid phosphatase	48.305	7.02	membrane; nucleus	metabolic process; regulation of biological process; transport

[1] Acc. No., UniProt accession number [2] MW, Molecular weight [3] pI, Isoelectric point
