# Supplementary figures and images for: Characterization of Proteins Regulated by Androgen and Protein Kinase a Signaling in VCaP Prostate Cancer Cells
Source: Biomedicines. 2021 Oct 6;9(10):1404. doi: 10.3390/biomedicines9101404 (PMC8533394; doi:10.3390/biomedicines9101404)

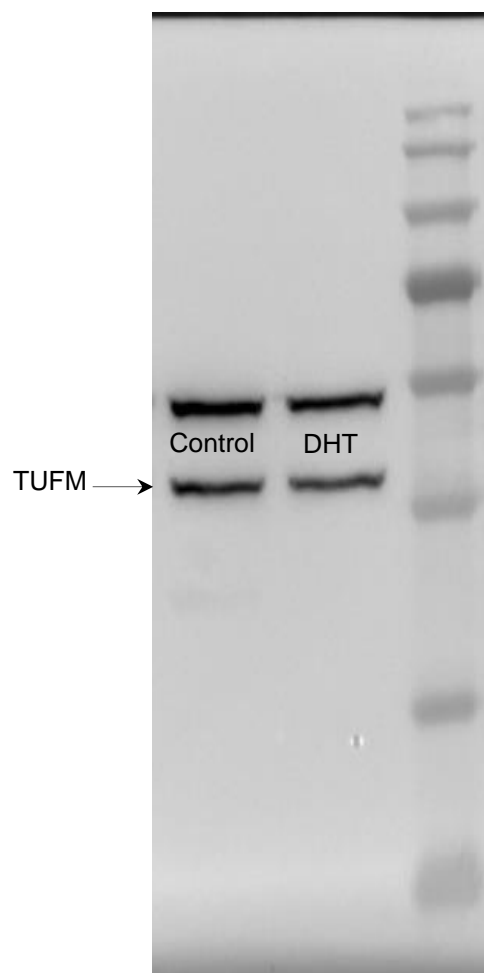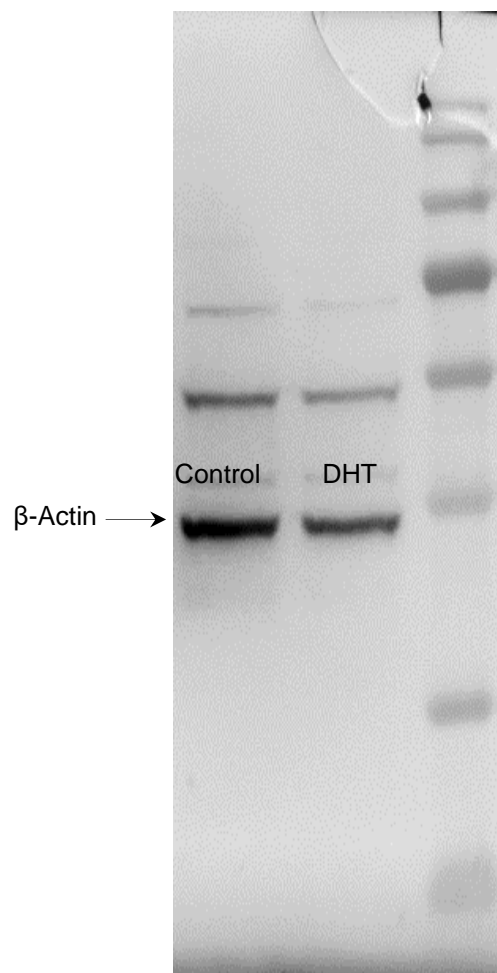

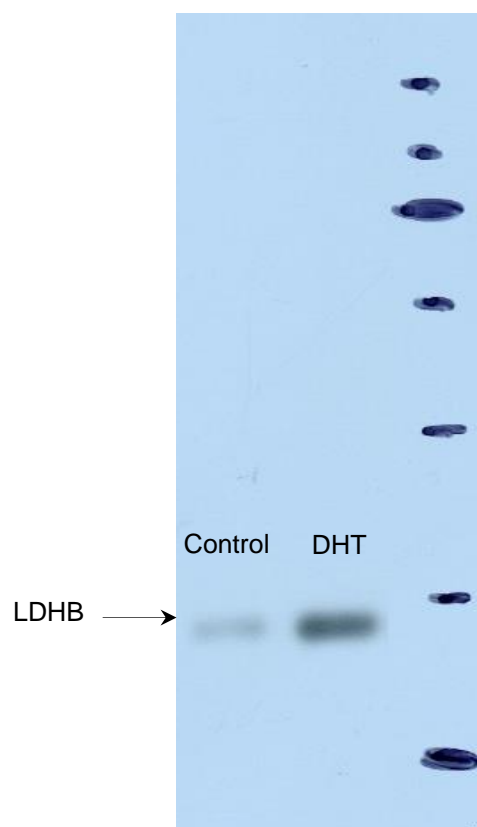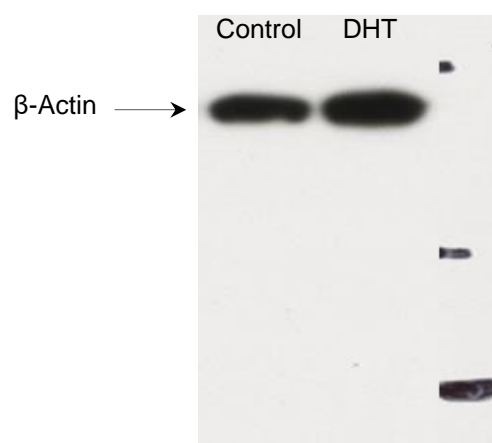

OXCT1 →

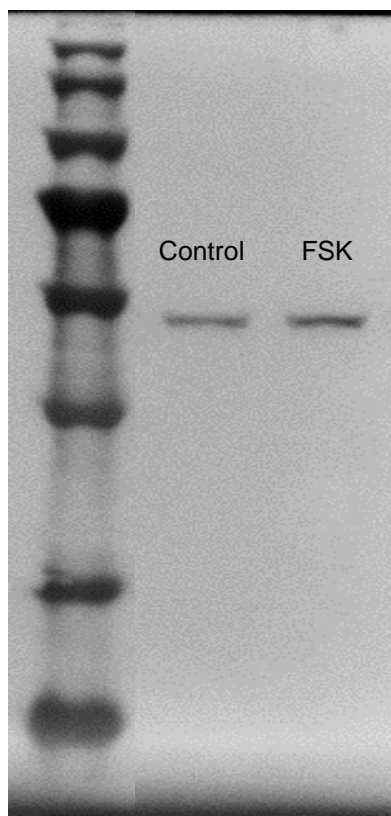

β-Actin →

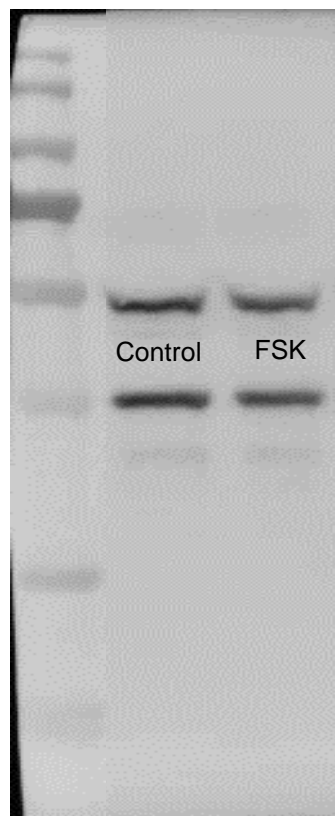

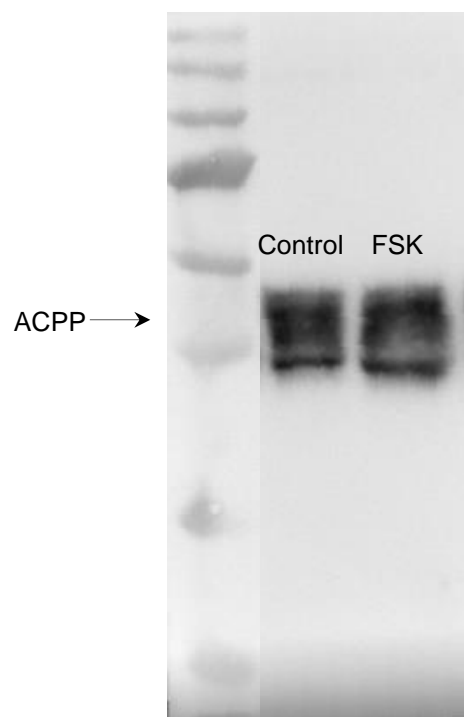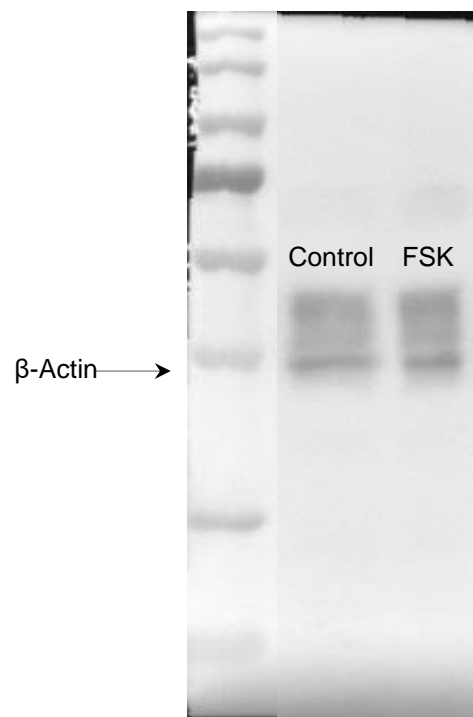

Supplement: Supplementary file 1 [file biomedicines-09-01404-s001.zip › WB_original images.pdf]

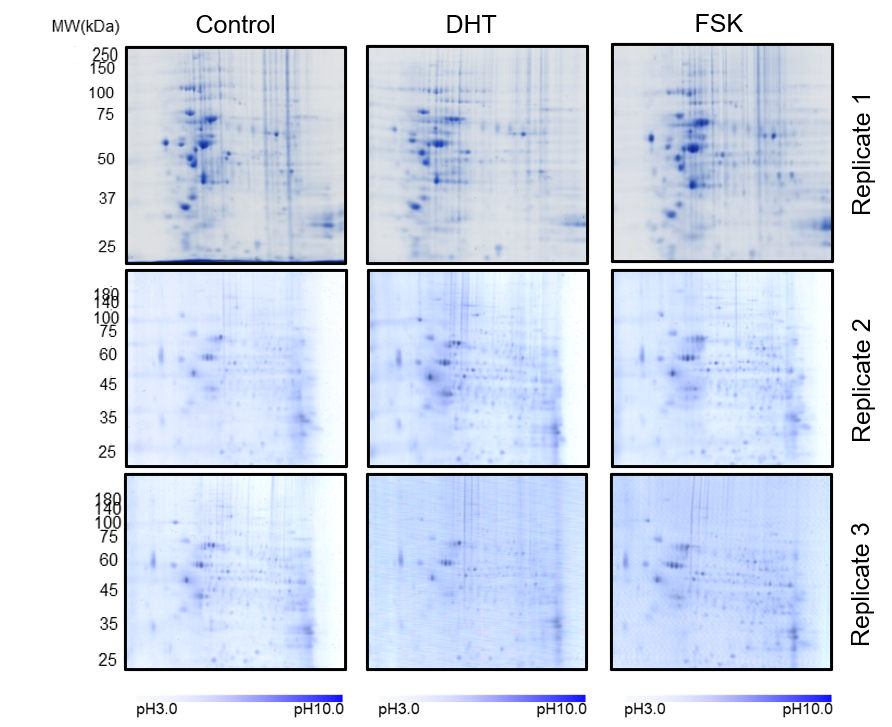

Supplement: Supplementary file 1 [file biomedicines-09-01404-s001.zip › Figure S1.tif]

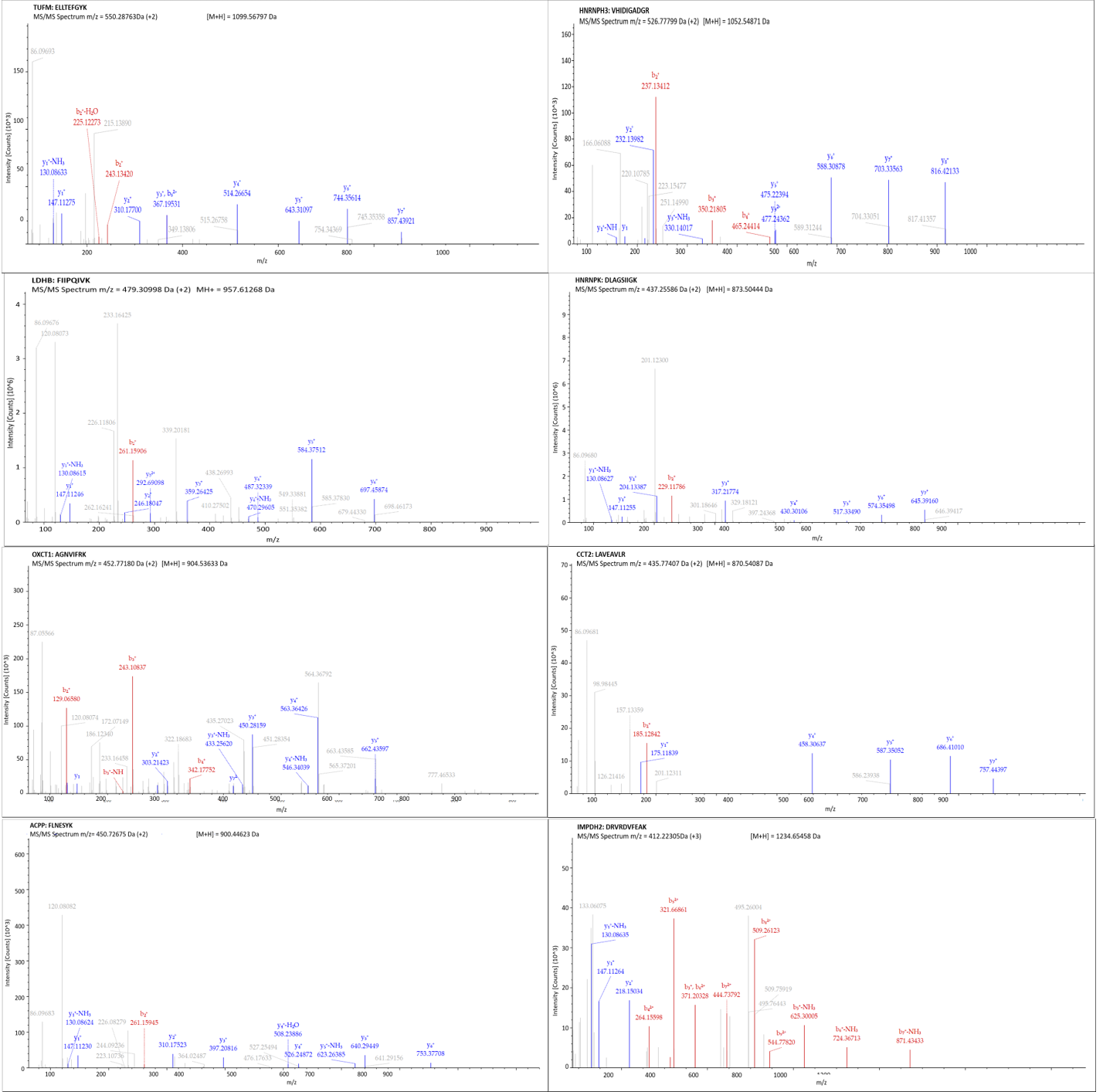

Supplement: Supplementary file 1 [file biomedicines-09-01404-s001.zip › Figure S2.tif]

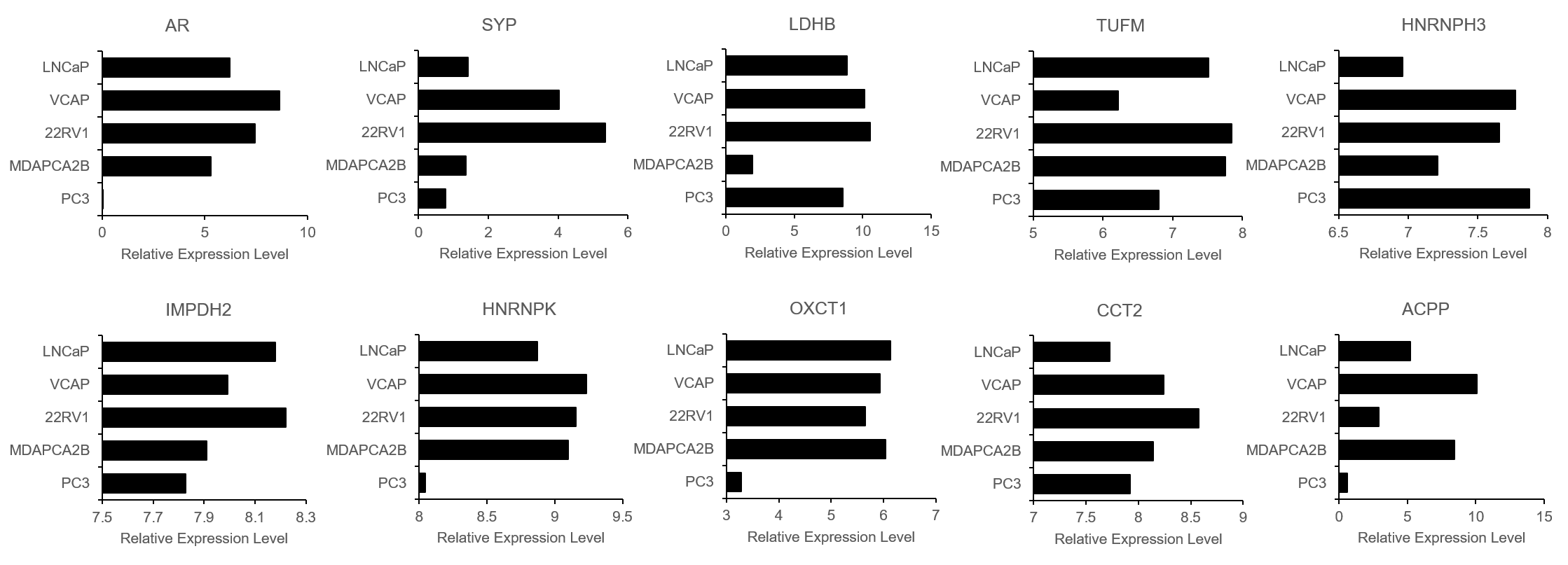

Supplement: Supplementary file 1 [file biomedicines-09-01404-s001.zip › Figure S3.tif]
